# DISMISS: detection of stranded methylation in MeDIP-Seq data

**DOI:** 10.1186/s12859-016-1158-7

**Published:** 2016-07-29

**Authors:** Umar Niazi, Kathrin K. Geyer, Martin J. Vickers, Karl F. Hoffmann, Martin T. Swain

**Affiliations:** 1 Institute of Biological, Environmental, and Rural Sciences (IBERS), Aberystwyth University, Penglais, Aberystwyth, Ceredigion SY23 3FG UK; 2 Imperial College London, NIHR HPRU in Respiratory Infections, Medical School Building, St Mary’s Campus, Norfolk Place, London, W2 1PG UK

**Keywords:** DNA methylation, MeDIP-Seq, BS-Seq, *Apis mellifera*, DISMISS, Epigenetics, Asymmetric, Galaxy

## Abstract

**Background:**

DNA methylation is an important regulator of gene expression and chromatin structure. Methylated DNA immunoprecipitation sequencing (MeDIP-Seq) is commonly used to identify regions of DNA methylation in eukaryotic genomes. Within MeDIP-Seq libraries, methylated cytosines can be found in both double-stranded (symmetric) and single-stranded (asymmetric) genomic contexts. While symmetric CG methylation has been relatively well-studied, asymmetric methylation in any dinucleotide context has received less attention. Importantly, no currently available software for processing MeDIP-Seq reads is able to resolve these strand-specific DNA methylation signals. Here we introduce DISMISS, a new software package that detects strand-associated DNA methylation from existing MeDIP-Seq analyses.

**Results:**

Using MeDIP-Seq datasets derived from *Apis mellifera* (honeybee), an invertebrate species that contains more asymmetric- than symmetric- DNA methylation, we demonstrate that DISMISS can identify strand-specific DNA methylation signals with similar accuracy as bisulfite sequencing (BS-Seq; single nucleotide resolution methodology). Specifically, DISMISS is able to confidently predict where DNA methylation predominates (plus *or* minus DNA strands – asymmetric DNA methylation; plus *and* minus DNA stands – symmetric DNA methylation) in MeDIP-Seq datasets derived from *A. mellifera* samples. When compared to DNA methylation data derived from BS-Seq analysis of *A. mellifera* worker larva, DISMISS-mediated identification of strand-specific methylated cytosines is 80 % accurate. Furthermore, DISMISS can correctly (*p* <0.0001) detect the origin (sense vs antisense DNA strands) of DNA methylation at splice site junctions in *A. mellifera* MeDIP-Seq datasets with a precision close to BS-Seq analysis. Finally, DISMISS-mediated identification of DNA methylation signals associated with upstream, exonic, intronic and downstream genomic loci from *A. mellifera* MeDIP-Seq datasets outperforms MACS2 (Model-based Analysis of ChIP-Seq2; a commonly used MeDIP-Seq analysis software) and closely approaches the results achieved by BS-Seq.

**Conclusions:**

While asymmetric DNA methylation is increasingly being found in growing numbers of eukaryotic species and is the predominant pattern observed in some invertebrate genomes, it has been difficult to detect in MeDIP-Seq datasets using existing software. DISMISS now enables more sensitive examinations of MeDIP-Seq datasets and will be especially useful for the study of genomes containing either low levels of DNA methylation or for genomes containing relatively high amounts of asymmetric methylation.

## Background

Methylated DNA immunoprecipitation sequencing, or MeDIP-Seq, is a common methodology used to study DNA methylation profiles within plant and animal genomes [1]. DNA methylation is increasingly being recognised as playing a central role in the regulation of gene expression and chromatin structure. Cytosine methylation is a particular type of DNA methylation that most often refers to the reversible addition of a methyl group to the carbon-5 position of the cytosine pyrimidine ring, resulting in the formation of 5-methylcytosine (5mC) modifications within double stranded DNA [2]. These epigenetic DNA modifications can be further classified based on the nucleobase context in which 5mC is observed. Double stranded DNA methylation primarily occurs if the methylated cytosine occurs within cytosine-guanosine dinucleotide sequences i.e. CG context (also known as CpG methylation where the phosphate bond between the nucleotides is explicitly represented as a ‘p’). This is due to nucleobase symmetry between sense (CG) and antisense (GC) DNA strands. However, methylation in a CG context is not always double-stranded; it can also occur on just a single strand. Single stranded (also known as asymmetric, strand-biased or hemimethylation) DNA methylation can additionally be detected if the 5mC is in a non-CG methylation context, i.e. a CH dinucleotide context where H = A, T or C [3–5].

Asymmetric DNA methylation is a common phenomenon of plant genomes [6]. For example it has been associated with methylation changes on retrotransposons during the development of pollen cells [7]. However, for many years it was thought to be absent in mammals where much of the initial focus was on the detection of symmetric CG methylation. Nevertheless, the presence of non-CG methylation in mammalian embryonic stem and induced pluripotent stem cells [8, 9], as well as somatic tissues, e.g. adult brain [9–11] is becoming more commonly observed. Although asymmetric DNA methylation might not have the same function as symmetric double-stranded methylation (i.e. in gene silencing), there is preliminary evidence implying a link between non-CG methylation and transcriptional regulation [11]. In contrast to symmetric CG methylation, cytosine methylation in a non-CG context is prevalent within gene bodies, particularly exons, rather than surrounding the transcriptional start site [3, 12]. There is substantial evidence suggesting that non-CG methylation serves a distinct function depending on the cell type. For instance, non-CG methylated regions are prevalent in undifferentiated cells such as embryonic stem cells [3, 8], as well as differentiated cells such as brain and skeletal muscle [9, 10]. Additionally, non-CG methylation is generally associated with a positive correlation to gene expression: this modification is thought to act as a repressive mark in the adult mammalian brain and appears to be crucial for neural function [11, 13]. DNA methylation studies of other animals (e.g. invertebrates) initially were hampered by assumptions translated from mammalian systems, such as the predomination of methylation occurring at CG dinucleotide sequences [14]. Recently, however, this view is being challenged with the honey bee, *Apis mellifera*, becoming an increasingly important invertebrate model for studying how DNA methylation (in diverse dinucleotide contexts) affects metazoan development, behaviour and gene splicing [15–17]. For example, Cingolani et al. [17] have described how biases in both software and experimental design towards studying CG methylation have led to DNA methylation in other dinucleotide contexts being poorly identified and functionally underappreciated. After correcting for such biases, they were surprised to discover 5-fold more CHH methylation than CG methylation in *A. mellifera*. They report that of all the cytosines present in CG, CHG, and CHH contexts in the genome, 2.5 % were methylated; of these, only 21 % of CG cytosines were symmetrically methylated with 0 % of CHH and 0.53 % of CHG cytosines symmetrically methylated. In total, symmetric methylation occurred in only 3.4 % of the methylated cytosine positions. Thus, asymmetric methylation predominates in this invertebrate species. Cingolani et al. further suggest that non-CG modifications might have a significant influence on the regulation of alternative splicing, and this highlights the need to detect and quantify strand-specific methylation in other metazoan organisms [17].

The current MeDIP-Seq library preparation procedure preserves strand identity, meaning that MeDIP-Seq reads contain both symmetric and asymmetric strand methylation signals that are detectable [18]. However, this information is not exploited by any of the currently available software packages (e.g. MEDIPS [19] or MACS2 [20]). It therefore follows that the current downstream analysis methodology for MeDIP-Seq data is not optimal as asymmetric DNA methylation is not detectable. The ability to study strand-specific DNA methylation signals would significantly increase the utility of MeDIP-Seq and lead to further insight into the function of asymmetric DNA methylation. We, therefore, have developed a new software (DISMISS; Detection of Stranded Methylation in MeDIP-Seq Data) to decompose MeDIP-Seq derived DNA methylation information into individual, strand-specific signals (i.e. identifying 5mC on plus, minus or both DNA strands).

We have used DISMISS to confidently assign strand-associated DNA methylation signals in MeDIP-Seq datasets derived from *A. mellifera.* We have also quantified how well DISMISS predicts strand-associated DNA methylation in *A. mellifera* when compared to data obtained from a technology that is able to resolve methylation signals at the nucleotide-level, namely BS-Seq (the sodium bisulfite conversion of DNA followed by sequencing [4]). By demonstrating how DISMISS can increase the resolution and sensitivity of MeDIP-Seq analysis of gDNA (e.g. allowing DNA methylation to be assigned to a specific DNA strand), we present an enabling methodology that can be used to more accurately study DNA methylomes.

## Implementation

### Strand conservation in MeDIP-Seq libraries

**Fig. 1:**
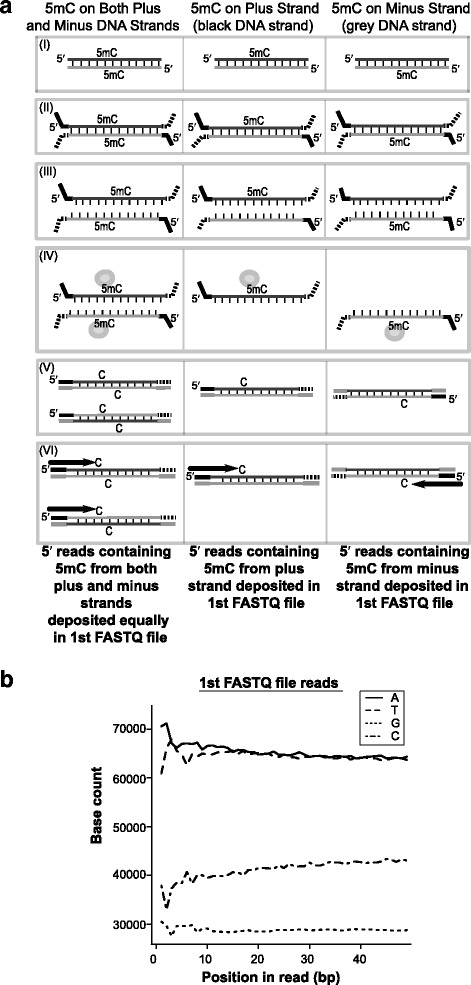
DISMISS utilises strand biases generated during MeDIP-Seq library preparation to detect strand-specific DNA methylation. **a** Outline of MeDIP-Seq library preparation. (I) Genomic 5mC can be present in three possible scenarios: (*left*) with 5mC found on both DNA strands, (*middle*) with 5mC found only on the plus DNA strand (black coloured strand) and (*right*) with 5mC found only on the minus DNA strand (*grey coloured* strand). (II) DNA is fragmented, end-repaired and the forked adapters ligated directionally to the DNA fragments – *solid black* (5′ adapter) and *dashed black* (3′ adapter); (III) DNA is denatured. (A-IV) MeDIP enrichment (*grey* spheres) selects single-stranded 5mC containing fragments; a sampling bias arises due to the selection of strands originating from DNA fragments containing 5mC on only a single strand (*middle* and *right*) – note the *black* adaptor is retained, identifying the strand origin of the selected fragment. (V) PCR library enrichment is performed (5mC lost during PCR amplification and replaced with C). (VI) *Black arrows* show sequencing of first mate reads (containing the MeDIP selected 5mC) occurring from the *black* adaptor. DNA fragments from all three scenarios are sequenced from both ends, with those originating from *black* and *grey* adaptors deposited in a single pair of first and second FASTQ files, respectively. Reads from the minus strand will tend to align to the genome sequence in reverse-compliment mode. **b** C to G ratio bias detected in a MeDIP-Seq FASTQ file. Base count per cycle plot for the first FASTQ file. The x-axis shows the base position in the read, while the y-axis shows the count of each nucleotide at that position. The A to T ratios do not show a bias, as is expected for a random library not selecting for either of these nucleobases. However, the C to G ratio demonstrates a clear bias due to the selection of 5mC containing fragments (in step A-IV)

The retrieval of strand identity during MeDIP-Seq analyses of genomes is possible due to sequencing adapter directionality [18]. The methodological step-by-step process of retrieving strand-specific information is illustrated in Fig. 1a. In part (I) (of Fig. 1 (a)), genomic 5mC can be represented by three possible scenarios: firstly, the cytosine on both DNA strands is methylated (symmetric), secondly the 5mC is found only on the plus DNA strand (asymmetric), and lastly when the 5mC occurs only on the minus DNA strand (asymmetric). During the first phase of library preparation (II), gDNA is fragmented, end-repaired and the forked adapters are ligated directionally. Subsequently, the gDNA is denatured (III) prior to the MeDIP 5mC immuno-capture step (IV), which leads to the selection of single stranded 5mC containing DNA fragments with the 5′ adaptor shown as a bold solid black line – the presence of this adaptor is important for resolving the strand specific methylation signal. Following the 5mC immuno-capture step, library preparation continues by performing a PCR amplification step (V), resulting in double stranded DNA fragments. No matter which of the three scenarios the original 5mC is found in, the sequencing of first mate reads is initiated from the 5′ adaptor (VI) – and due to the previous selection of asymmetrically methylated fragments (plus or minus strands only) in step (IV) this creates a disparity between the numbers of C nucleobases and G nucleobases in the first FASTQ file. The strand origin of the original fragments may be recovered by aligning the sequenced reads to the genome sequence: reads containing 5mC from the minus strand will align in reverse-compliment mode.

The disparity in the G to C ratio of the MeDIP reads can be readily observed using quality control software for high-throughput sequencing data (Fig. 1b). For instance, software packages such as FastQC (http://www.bioinformatics.babraham.ac.uk/projects/fastqc/) are commonly used to assess a number of read metrics, including the ‘Base frequency per cycle’. In a typical random gDNA library, the ratio between the counts of A and T nucleobases should be approximately equal, as should the G to C ratio. In a MeDIP-enriched gDNA library, however, the plot will clearly show different counts for C and G, as shown in the ‘Base frequency per cycle plot’ of Fig. 1b. This QC abnormality is more pronounced in cases where DNA methylation is primarily found in an asymmetric context.

### MeDIP-Seq strand separation (DISMISS)

If the MeDIP-Seq library has been prepared following the methodological outline described in Fig. 1 (i.e. strand information is conserved), then this information can be exploited by DISMISS to assign 5mC strand specificity to each peak called by a peak caller (e.g. MACS2). Thus, each DNA methylation peak can be classified into one of the three classes: 1) Minus Stranded, 2) Plus Stranded or 3) Peaks on Both Strands. The methods required to perform this classification are implemented in the DISMISS software and are described in the following paragraphs.

The count of MeDIP reads mapping to a genomic location can be modelled as a Poisson distribution [21]. Let *Y* be the number of independent reads mapping to a genomic location that has previously been identified as a peak region by MACS2. The distribution of *Y* is assumed to be Poisson with the parameter lambda (λ):

$$ Y\sim Poi\left(\lambda \right) $$

The reads mapped to the MACS2 peak region are divided into two sub events representing the number of independent reads mapping to each of the two strands, *Y*_*plus*_ and *Y*_*minus*_. These sub events are also distributed according to a Binomial distribution:

$$ {Y}_{plus}\sim Poi\left(\uplambda \uptheta \right)\ \mathrm{and}\;{Y}_{minus}\sim Poi\left(\uplambda \left(1-\uptheta \right)\right), $$

where *θ* is a fraction given by *Y*_*plus*_/*Y*.

The goal of the analysis is to find out if there are integer values of *λ* that are plausible for both *Y*_*1*_ and *Y*_*2*_. The likelihood function gives the probability of seeing a Poisson distributed value *Y*, given a rate *λ*:

1 $$ \boldsymbol{l}\left(\uplambda \right)= Pr\left[\left.\mathrm{Y}\right|\uplambda \right] $$

For all the plausible values of *λ* there is a unique value of *λ* that maximises the probability given by Eq. 1, called the maximum likelihood estimate of *λ*:

2 $$ \Lambda = argma{x}_{\uplambda}\boldsymbol{l}\left(\uplambda \right) $$

In addition to finding the maximum likelihood estimate, we also want to know what other values of *λ* have a reasonably high likelihood in order to capture the randomness of the biological process. A likelihood set is a set of *λ* values that explain the data in a statistically significant manner:

3 $$ \mathrm{L}{\mathrm{S}}_{\upalpha}\equiv \left\{\uplambda :\left[\boldsymbol{l}\left(\uplambda \right)/\boldsymbol{l}\left(\Lambda \right.\right]\ge \upalpha \right\} $$

Where *α ∈ (0,1)* is the cut-off value and we frequently use *α ≈ 0.1* for convenience and custom. Further details of the Poisson distribution and the comparison of distributions using likelihood ratios are thoroughly explained elsewhere [22].

For each peak region with *Y* reads, the decision to assign a class is performed in the following manner. Given that *Y*_*plus*_ are the number of first mate reads aligned to the plus strand and *Y*_*minus*_ the number of first mate reads aligned to the minus strand, then a set of integer values from and including *Y*_*plus*_ to *Y*_*minus*_ that are plausible values of *λ* to explain the data are chosen. Using this set of *λ* values and Eq. 3, two likelihood sets are generated. If the intersection of these two sets is not empty, then there are values of *λ* that are plausible for both *Y*_*plus*_ and *Y*_*minus*_ and the peak is assigned to class ‘Peaks on Both Strands’. Otherwise the intersection of the sets is empty and the peak will be assigned as single-stranded: if *Y*_*plus*_ is greater than *Y*_*minus*_ then the peak is assigned to class ‘Plus Stranded’, else the peak is assigned to class ‘Minus Stranded’.

For example, if the number of reads mapping to the peak identified by MACS2 is given by *Y = 100*, with the number of reads mapping to the two strands comprising this peak given by *Y*_*plus*_ *= 60* and *Y*_*minus*_ *= 40*, then the set of plausible values for *λ* (from and including *Y*_*plus*_ to *Y*_*minus*_) is given by {40, 41,…, 59, 60} and *θ* will be 60/100 = 0.6. Using the Poisson distributions for *Y*_*plus*_ and *Y*_*minus*_ and Eq. 1, the likelihood for each value of *λ* in this set is calculated and Eq. 2 is used to select two values of *λ* that maximise the likelihood for each plus and minus strand. Furthermore, using Eq. 3, two likelihood sets (LS) containing plausible values of *λ* (i.e. α > = 0.1) are generated: LS_plus_ = {45, 46, …, 59, 60} and LS_minus_ = {40, 41, …, 54, 55}. The intersection of these two sets is not empty, hence there are common values of *λ* with a high likelihood for the observed number of reads *Y*_*plus*_ and *Y*_*minus*_ and the peak is assigned to both strands.

### Data sets used and analysis procedures

**Fig. 2:**
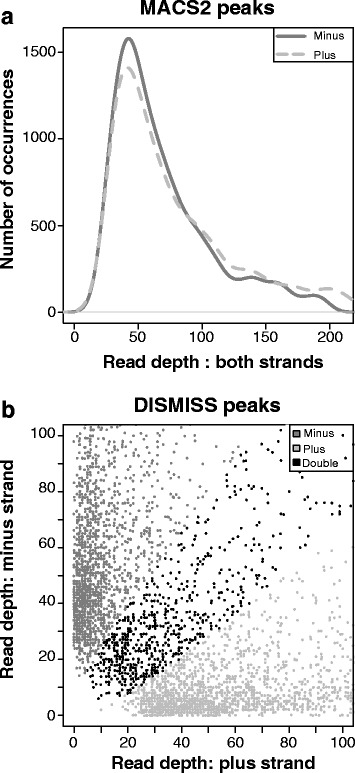
DISMISS predictions of the DNA strand on which the 5mC occurs are not influenced by read depth. **a** MACS2 peaks. The frequency of occurrence of peaks, with a certain depth of first mate reads, on the plus and minus strands. The minimum read depth is 12 first mate reads. **b** DISMISS peaks. The depth of first mate reads on the plus strand is plotted against the depth of first mate reads on the minus strand. The predictions made by DISMISS are indicated in grey scale and marked on the graph; they show well defined linear boundaries. The minimum read depth is 12 first mate reads

**Fig. 3:**
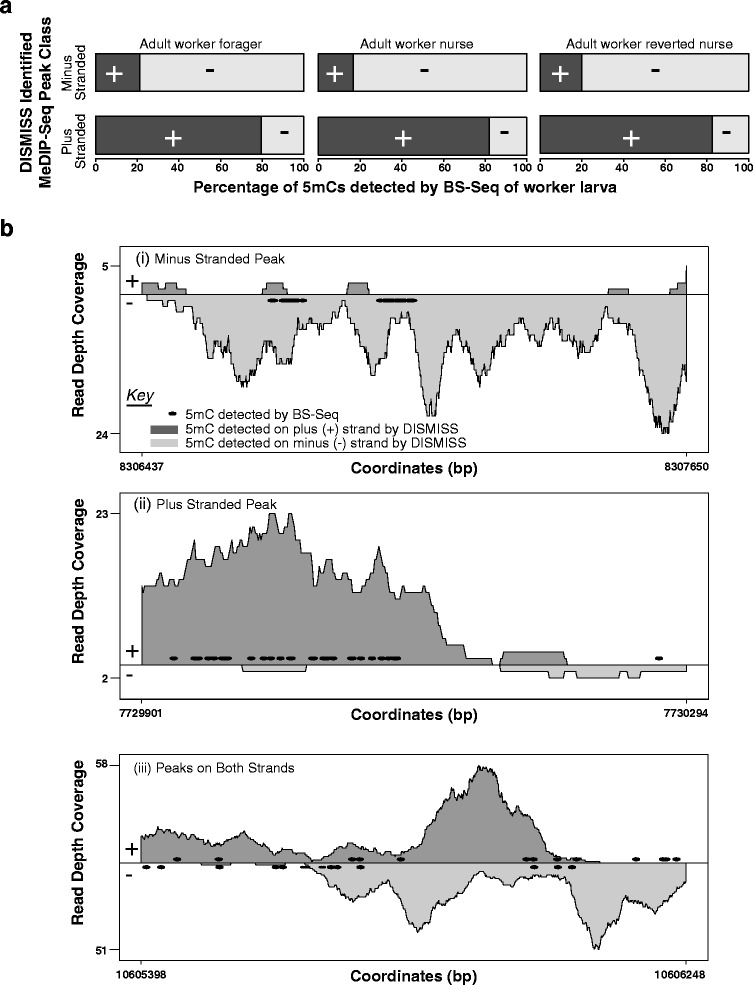
DISMISS interrogation of MeDIP-Seq datasets can accurately predict the DNA strand where 5mC occurs. A comparative analysis of 5mC detected by DISMISS interrogation of MeDIP-Seq data with 5mC detected by BS-Seq is illustrated (see the Implementation subsection on data sets for SRA identifiers) **a** Despite originating from different developmental forms, 5mC detected by DISMISS analysis of MeDIP-Seq datasets and BS-Seq datasets are concordant. Each horizontal bar represents a DISMISS quantified peak class (plus stranded or minus stranded) of MeDIP-Seq data. From all of the DISMISS predicted peak regions in a class, the percentage of 5mCs on the Plus and Minus strands was calculated from the BS-Seq dataset. Each bar shows the percentage of 5mC identified by BS-seq on either plus strands (+, *Black*) or minus strands (−, *Grey*). The DISMISS predicted ‘Minus Stranded’ and ‘Plus Stranded’ peaks predominantly contain BS-Seq identified 5mCs on either Minus or Plus strands, respectively. **b** Loci-specific examples of 5mC detected by DISMISS analysis of MeDIP-Seq datasets, with the corresponding BS-Seq predictions, over three genomic regions. Three randomly selected regions from the *A. mellifera* genome (assembly NC_007070.3), corresponding to DISMISS predicted peak classes (i, Minus stranded 5mC; ii, Plus stranded 5mC; and iii, 5mC peaks found on both strands) are indicated. The x-axis shows the NC_007070.3 base pair coordinates of each selected region. The first mate read coverage for each base using DISMISS-assessed MeDIP-Seq alignment data is shown along the y-axis. The y-axis is divided into two parts by a central line where the dark-grey coverage above the line represents reads predicted to map to the Plus strand and the *light-grey* coverage shows reads predicted to map to the Minus strand (see key). The *black dots* above the line indicate BS-Seq identified 5mCs found on the Plus strand, while those below the line show BS-Seq identified 5mCs found on the Minus strand

**Fig. 4:**
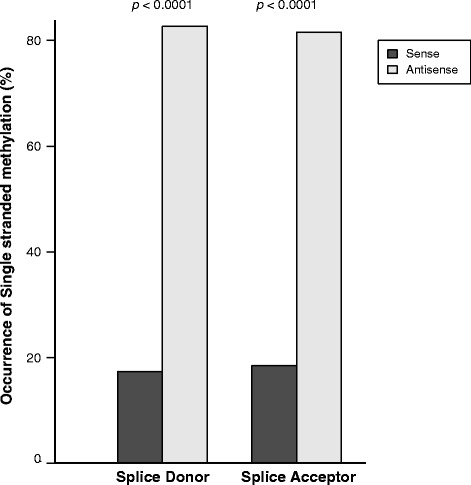
DNA methylation is accurately predicted by DISMISS over gene splice sites in *A. mellifera*. The x-axis shows two genomic regions representing intron splice donor and acceptor sites. The y-axis shows the percentage of single stranded MeDIP-Seq peaks (plus stranded and minus stranded), predicted by DISMISS, on the sense (*dark grey*) and antisense (*light grey*) sides of these regions. DISMISS detected more DNA methylation on the antisense sides of the splice acceptor and donor sites, compared to the sense side, which is statistically significant (using Two tailed proportion test) with *p* values less than 0.0001 (see the Implementation subsection on data sets for SRA identifiers)

**Fig. 5:**
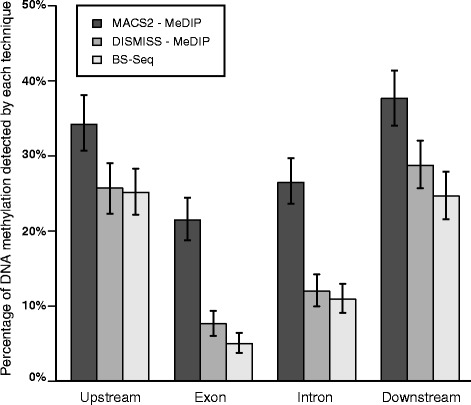
DISMISS detection of DNA methylation in MeDIP-Seq datasets approaches the resolution obtained by BS-Seq analysis. A MeDIP-Seq dataset was analysed by MACS2 and DISMISS. The proportion of DNA methylation found in four different genomic regions (2 KB upstream of the gene’s 5′ end, exons, introns and regions 2 KB downstream of the gene’s 3′ end) was deduced and compared to the proportion of DNA methylation signal (in these same regions) detected in a BS-Seq dataset (see the Implementation subsection on data sets for SRA identifiers)

MeDIP-Seq data derived from *A. mellifera* [23] consisted of adult worker nurse with Sequence Read Archive (SRA) number SRR850130 (used in Fig. 2a), adult worker forager with SRA number SRR850131 (used in Fig. 3a) and adult worker reverted-nurse with SRA number SRR850132 (used in Figs. 1b, 2a, b, 3a, b, 4 and 5). The BS-Seq data originated from *A. mellifera* worker larvae with SRA number SRX101302 [24] (used in Figs. 3a, b, and 5). All data sets used in this study were retrieved using the SRA software toolkit provided by the NCBI (National Center for Biotechnology Information).

#### MeDIP-Seq and BS-Seq data analysis

The MeDIP-Seq FASTQ files were aligned to the *A. mellifera* genome [25] using Bowtie2 [26]. SAMtools [27] was subsequently used to remove aligned reads with a mapping quality below 10 as well as to eliminate PCR and optical duplicates, following the protocol suggested by Taiwo et al. [5]. The peak calling to identify peak regions (DNA methylation windows) was performed using MACS2 (with no control and options –m 5 50 –g 2.3 × 10^8^ –q 0.01 for the ‘callpeaks’ module) [20]. The *A. mellifera* BS-Seq data set used in Fig. 3 was processed by Bismark [28] using default options and bowtie (version 1) to identify 5mCs; only 5mCs covered by at least three methylated reads were included in the analysis.

Additionally, to enable the *A. mellifera* datasets to be viewed in the Integrative Genomics Viewer (IGV) [29] various BedGraph files were generated (and converted to the TDF format required by IGV). These files include stranded output for the BS-Seq 5mC calls in all methylation contexts, the coverage of the MeDIP reads including a separate track for the first mates and the stranded calls made by DISMISS for each of the three *A. mellifera* developmental stages analysed. All of these files are available (along with the DISMISS software) at http://uhkniazi.github.io/dismiss.

#### Annotation

The genome and annotation information for *A. mellifera* (version 4.5) [25, 30] were obtained from the NCBI.

#### Counting overlaps of genomic features

A location in the genome can be represented as a set of start and end coordinates, a strand (plus, minus or both) and sequence name (chromosome or scaffold name). Two locations or features are overlapping if they have the same sequence name, compatible strand pairing and overlapping coordinates. The Bioconductor package GenomicRanges provides this data structure to handle genomic features along with various utility functions to perform operations on these features. In our analysis, the counting of overlapping features was performed using the Bioconductor *countOverlaps* function:

4 $$ C= CountOverlaps\left(Q,S\right) $$

Where *Q* is the query parameter (features of interest); *S* is the subject parameter (features over which counting is performed); *C* is a set of integer values of length equal to Q, giving counts of overlaps of *Q* and *S*.

## Results

Here, for the first time, we describe a tool to improve the resolution of 5mC analysis using MeDIP-Seq, which allows for the accurate determination of symmetric vs asymmetric DNA methylation. Previously, the accuracy of MeDIP-Seq has been investigated via comparison with BS-Seq and a good concordance in CG methylation between these technologies for symmetric methylation has been demonstrated [4]. Following this strategy, we demonstrate the accuracy of DISMISS by performing a comparison of 5mC strand-specific predictions made on an existing MeDIP-Seq data set to 5mC sites verified in an approximately equivalent BS-Seq data set, both derived from an invertebrate genome with a relatively high level of asymmetric methylation, the honeybee (*A. mellifera*).

### Asymmetric methylation prediction: investigating potential sources of bias

DISMISS was used to identify strand specific methylated DNA regions in existing MeDIP datasets derived from three adult worker life stages: nurse, forager, and reverted nurse (note that for the worker caste, bees first develop from larva to nurse, then from nurse to forager, and finally a forager may revert back to the nurse stage). Depending on the life stage, MACS2 identified approximately 34,000 to 37,000 methylated regions within the whole honey bee genome; DISMISS identified 80 to 85 % of those as being single-stranded regions with the remaining 15 to 20 % being double-strand regions. Plots showing the distribution of strand read depth from MACS2 peaks are given in Fig. 2a. In order to demonstrate that strand decomposition of the MACS2 peaks by DISMISS is not influenced in a negative way by read depth, the distributions of read depths for plus, minus and double stranded peaks are shown in Fig. 2b. The figure shows a clear demarcation of the stranded categories. To investigate the possibility of bias arising in peak regions that are wide enough to include both single and double stranded methylation we performed the following tests. Based on the quantiles of the peak widths, we split the peak regions into three groups with the following widths: group 1: from 26 to 439 bp; group 2: from 440 to 770 bp; and group 3: from 771 to 2500 bp. Groups 1, 2 and 3 contain 50, 45 and 5 % of the peaks, respectively. Group 3 has the widest peaks and would therefore be most likely to contain a mix of double and single-stranded peaks. In each of these groups we counted the numbers of plus stranded, minus stranded and double stranded peaks. These counts were used in a contingency table to test the null hypothesis that the distribution of plus, minus and double stranded peaks is independent of peak width: a Chi-squared test gave a *p*-value of 0.17. Thus, there is a slight bias in the widest peak regions, but the trend is not statistically significant.

### Asymmetric methylation prediction: concordance between DISMISS and BS-Seq

Using BS-Seq data from an additional life stage (worker larvae), Bismark ascertained that 6, 14 and 42 % of the 5mCs in CG, CHG, and CHH contexts were methylated, respectively. Thereafter, only regions identified by DISMISS were considered in this analysis. These regions were centred on the peaks identified by MACS2 from the MeDIP data and ranged from about 260 to 2500 bp in length with an average size of about 430 bp. The 5mCs identified within these regions by Bismark (from the BS-Seq) were separated into the fractions (percentages) present on each of the plus and minus strands (Fig. 3), and counted using Eq. 4. To independently validate the DISMISS predictions, a *p*-value was calculated using a paired two-tailed *t*-test and was performed three times: one for each peak class from the DISMISS prediction (plus or minus stranded, or on both i.e. double-stranded) to test the null hypothesis that the number of 5mCs on the plus and minus strands are equal. Figure 3a summarises the concordance between the two approaches within the strand-associated DNA methylation regions identified by DISMISS, across the whole honeybee genome. Within the DISMISS predicted ‘Minus Stranded’ DNA methylation regions, more BS-Seq identified 5mCs were classified on the minus strand (*p*-value <0.0001). Similarly, in the DISMISS predicted ‘Plus Stranded’ DNA methylation regions, more BS-Seq identified 5mCs were detected on the plus strand (*p*-value <0.0001). Within the regions predicted by DISMISS to contain methylation on both strands (‘Double Stranded’), there were approximately equal numbers of BS-Seq identified 5mCs detected on both plus and minus strands (no statistically significant strand bias; *p*-value >0.05). Exemplar genomic regions selected to show instances of good concordance between BS-Seq and MeDIP for each of the three cases of strand-associated DNA methylation are illustrated in Fig. 3b. The y-axis depicts the first mate read coverage from the MeDIP-Seq alignment data and the x-axis represents the 5mCs observed in BS-Seq data as black dots (on either plus and/or minus strands along with base pair coordinates). For a more detailed overview of the concordance between BS-Seq, MeDIP, and DISMISS at specific loci we have prepared the appropriate files (in BedGraph and GFF format) as tracks for viewing in a genome viewer such as IGV (Integrative Genomics Viewer) [29] (see the “Availability of data and materials” section).

### Single stranded DNA methylation is asymmetrically distributed on sense and antisense strands

Using honeybee BS-Seq data, Cingolani, P. et al. have reported asymmetrical distribution of non-CG methylation at gene splice sites and have speculated about the role that this type of DNA methylation plays in alternative mRNA splicing [17]. Although we cannot match the nucleotide-level resolution of BS-Seq, here we test the functionality of DISMISS in identifying the presence of 5mC signals in either splice donor or splice acceptor sites in *A. mellifera* MeDIP-Seq data*.* A genomic feature such as a splice donor site can be present on either strand of the DNA duplex: the sense side is the strand on which the feature is present, which can be either the plus or minus strand and the antisense side is always the opposite strand. The number of single stranded DNA methylation signals (predicted by DISMISS) on the sense and antisense sides of each splice site were counted using Eq. 4. To independently validate the DISMISS predictions, a *p*-value was calculated using a two tailed proportion test [31] (prop.test in R), to test the null hypothesis that the proportion of single stranded DNA methylation on the sense and antisense sides of the feature are equal. In Fig. 4, we show that DISMISS-mediated DNA methylation predictions derived from MeDIP-Seq datasets indicate significantly greater (*p*-value <<0.0001) levels of DNA methylation are found on the antisense strand compared to the sense strand at genomic splice junctions in adult worker reverted-nurse samples. This observation is consistent with those made by Cingolani, P. et al. using BS-Seq [17]. Importantly, this is the first time that splice junction DNA methylation signals have been resolved from MeDIP-Seq datasets and demonstrates that DISMISS can play an important role in understanding the function of these genomic modifications during mRNA splicing.

### Comparison of DNA methylation in gene regions

The detection of DNA methylation over four distinct genomic features in the honeybee genome was subsequently compared using three different analysis strategies: MeDIP-Seq data processed by MACS2; MeDIP-Seq data processed by MACS2 and strands assigned by DISMISS; and BS-Seq data identified 5mCs using Bismark. The four genomic features analysed were upstream loci (defined as 2 KB upstream of the gene’s 5′ end), exons, introns, and downstream loci (defined as 2 KB downstream of the gene’s 3′ end). Genomic features occur in the genome with different degrees of abundance, for instance there is about three times more intronic sequence than exonic sequence in the *A. mellifera* genome [30]. In order to adjust for this variation we used Eq. 4 to count, per 1000 instances of a particular genomic feature, the number of overlaps between the methylation signal and that feature: the posterior distribution of DNA methylation signal over the four genomic features was modelled as four gamma-distributed variables using a conjugate Poisson sampling model. In Fig. 5 the counts per 1000 instances have been converted to percentages, and error bars show 95 % confidence intervals that were calculated using simulation [32]. Fig. 5 shows that the distribution of detectable MeDIP-Seq signal converges towards the BS-Seq signal after assigning strands to the methylated regions using DISMISS. These results demonstrate that DISMISS analysis of MeDIP-Seq datasets improves the resolution of detecting stand-associated DNA methylation signals (a stepwise improvement over just using MACS2) and offers a competitive alternative to BS-Seq for global methylome studies.

## Discussion

While a variety of sequencing-based methods for genome-wide DNA methylation profiling have been developed [4, 33, 34], bisulfite sequencing (BS-Seq) and methylated DNA immunoprecipitation sequencing (MeDIP-Seq) strategies are the most commonly used. BS-Seq results in single nucleobase classification of symmetric (cytosine methylation on both strands, CG) or asymmetric (cytosine methylation on plus or minus strand, CH) DNA methylation [33]. Nevertheless, it suffers from limitations that include incomplete bisulphite conversion of cytosines into uracils leading to false positives, possible PCR bias due to preferential amplification of methylated (C-rich) or unmethylated (T-rich) versions of the template, bisulfite conversion of methylated cytosines to uracils (over-treatment) leading to false negatives and, finally, the inability of sodium bisulfite to convert cytosines into uracils within repeat sequences forming snap-back structures [17, 33, 35, 36]. Furthermore, the sensitivity in detecting DNA methylation when using BS-Seq is generally a function of sequencing depth, which makes it relatively expensive to achieve sufficient resolution for detecting 5mC in poorly methylated genomes [33]. All of these reasons could explain why previously BS-Seq experiments failed to detect DNA methylation in *Drosophila melanogaster* [37] when liquid chromatography-tandem mass spectrometry (LC-MS) clearly demonstrated its presence – estimating that 0.034 % of cytosines were methylated [38].

In contrast, MeDIP-Seq has several advantages over BS-Seq in characterising DNA methylomes. As MeDIP-Seq is an enrichment-based technique, methylated DNA fragments are preferentially sequenced, resulting in greater sequencing depth of regions containing 5mC at an over-all lower total cost per genome [39]. Additionally, MeDIP-Seq can accurately detect 5mC in repeat regions and does not require the same amount of input gDNA for sample analysis in comparison to BS-Seq [5, 34, 39]. Therefore, if single-base 5mC resolution is not required, or the organism under study contains low DNA methylation levels, then MeDIP-Seq provides an excellent choice for global DNA methylome studies [38].

In terms of detecting methylation signals in genomes, MeDIP-Seq and BS-Seq generally show good concordance. Harris et al. compared two bisulphite-based techniques: MethylC-Seq and reduced representation bisulphite sequencing, RRBS (that reduces the proportion of the genome analysed to regions with high CG content) to two enrichment-based techniques (including MeDIP-Seq). They demonstrated that the assessment of DNA methylation in human embryonic stem cells by all four methods was 97 % concordant, using binary DNA methylation calls [4]. However, these techniques produced divergent results in terms of CG coverage, resolution, and quantitative accuracy. In particular, enrichment based techniques such as MeDIP-Seq lacked precision when quantifying DNA methylation levels due to the need to analyse genomic regions by averaging the number of CGs covered by variable numbers of reads in windows hundreds of base-pairs long.

Whilst exploring the greater resolution afforded to MeDIP-Seq studies by DISMISS, we had to focus on loci of methylation rather than the nucleotide-level resolution that is possible with BS-Seq. Additionally, we were unable to find any whole genome data sets that directly compare BS-Seq with MeDIP-Seq. Therefore, potentially confounding factors to our comparison between BS-Seq and MeDIP-Seq include the fact that the datasets used were derived from different laboratories, populations, and honey-bee life stages (for instance BS-Seq from larvae versus MeDIP-Seq from three different types of adult workers in Fig. 3). Nonetheless, despite originating from different developmental forms, overall analysis across the whole genome shows that the stranded predictions of 5mC made by DISMISS on MeDIP-Seq datasets are highly correlated with 5mCs detected within the BS-Seq datasets.

Our results demonstrate that by utilising the strand information present in MeDIP-Seq data sets, DISMISS can improve the resolution of the DNA methylation signal retrieved by other software packages (e.g. MACS2). Specifically, MeDIP-Seq data analysed by MACS2 and DISMISS achieves better concordance with BS-Seq than data analysed by MACS2 alone (Figs. 3 and 5). Furthermore, by applying DISMISS to MeDIP-Seq datasets, improved resolution of strand-associated DNA methylation signals can be achieved revealing biological features hidden in previous analyses (Fig. 4). Finally, as DISMISS takes advantage of a standard laboratory protocol for generating MeDIP-Seq libraries and acquiring data – there is no need to modify these for implementation.

## Conclusions

The standard laboratory protocol for MeDIP-Seq, together with DISMISS, offer a unique way to explore DNA methylation in either lowly methylated genomes where BS-Seq may fail to detect methylation, or in genomes that contain relatively high amounts of asymmetric methylation. We believe DISMISS is a significant contribution to the field of epigenetic data analysis and to facilitate its use by the community, we have provided both the software, and complete Galaxy [40–42] workflows for both DISMISS as a stand-alone application and for DISMISS embedded within a complete MeDIP-Seq data analysis.

## Abbreviations

BS-Seq, sodium bisulfite conversion of DNA followed by sequencing; MeDIP-Seq, methylated DNA immunoprecipitation sequencing
